# Reconstruction and validation of ground motions across dip-slip faults: an application to response analysis of a long-span suspension bridge

**DOI:** 10.1038/s41598-024-54558-z

**Published:** 2024-02-16

**Authors:** Hongyu Jia, Wei Cheng, Kang Jia, Yikun Zhai, Shixiong Zheng

**Affiliations:** 1 School of Civil Engineering, Southwest Jiaotong University, Chengdu, 610031 China; 2 State Key Laboratory of Bridge Intelligent and Green Construction, Ministry of Education, Chengdu, 610031 Sichuan China

**Keywords:** Dip-slip fault, Fling-step effect, Directivity effect, Fault-crossing suspension bridge, Fault rupture permanent surface displacement, Civil engineering, Natural hazards

## Abstract

Recent seismic events have unequivocally highlighted the susceptibility of fault-crossing bridges to the synergistic effects of ground surface vibrations on either side of the fault plane and the tectonic dislocations arising from fault-induced surface ruptures. This study delineates both seismic and parametric response analyses of fault-crossing suspension bridges, employing a straightforward yet efficacious method for simulating desired ground motions near fault-rupture zones. Herein, we introduce a user-friendly method to incorporate predicted fault-induced displacements, accounting for both fling-step and directivity effects, into processed ground motion chronologies, enabling the generation of dip-slip fault ground motions. The accuracy and efficacy of the proposed method are affirmed by juxtaposing the generated ground motions with the observed ones (MGM). An exhaustive parametric analysis, addressing factors like fault-crossing location, fault-crossing angle, and frequency components of fault-crossing ground motions, of a suspension bridge over a rupture fault, is executed using the fashionable ANSYS software. This study provides clear and specific guidelines for the seismic design of suspension bridges traversing rupture faults.

## Introduction

In the past few decades, there has been a tremendous expansion in the construction of railways and highways in China, notably in the seismically active regions of the southwest. Given the extensive network of potential active faults and the widespread construction and planning of bridges in earthquake-susceptible areas, it becomes infeasible to consistently avoid the construction of fault-crossing bridges in the light of national or local codes^1–3^. This perspective takes into account a myriad of factors, including topography, road planning, project expenses, construction timelines, and the demands of regional economic growth^4–7^. An escalating number of fault-crossing bridges have either been constructed or are under construction in the southwestern region of China^8^, such as the Puqian Approach Bridge in Hainan Province^9^. Initial investigations into fault-crossing bridges have aimed to establish clear seismic design guidelines. However, based on the authors' knowledge, these efforts, primarily centered on beam bridge structures, inadequately address the intricacies of long-span cable-supported bridges, especially suspension bridges, crossing rupture faults^10^.

It is widely recognized that during an earthquake, active faults can rupture and separate swiftly, leading to ground vibrations and simultaneous ground surface fractures. These fractures manifest as fault offsets, surface rupture permanent displacements, permanent translations, tectonic offsets, and fault dislocations^11,12^. The seismic behavior of fault-crossing bridges is influenced both by ground shaking on each side of the fault plane and by fault-rupture displacements ranging from tens of centimeters to several meters^13,14^. Based on structural dynamics theory, both fault-rupture displacement and ground shaking collaboratively dictate the dynamic responses of fault-crossing bridges. Specifically, they impact the quasi-static and relative dynamic terms in the structural vibration equation respectively^15–17^. Notably, Yang et al.^15^ highlighted variances in the relative contributions of quasi-static components and dynamic components to the overall bridge responses, differentiating between ordinary and seismically isolated bridges. Furthermore, faulting-induced surface rupture displacement stands out as a distinct differentiation in seismic excitation modes between fault-crossing and non-fault-crossing bridges. Consequently, the faulting-induced surface rupture displacement, which impacts the responses of fault-crossing bridges via the quasi-static term in natural vibration equations, plays a critical role in shaping the seismic behavior of bridges adjacent to active seismic faults^18,19^. The long-span suspension bridge, recognized as the most flexible bridge system for its span length, exhibits minimal sensitivity to the quasi-static term in the vibration equation^8^. Long-span suspension bridges excel in accommodating fault dislocations. Therefore, due to their capability to adapt to extensive fault rupture displacements, long-span suspension bridges are chosen to traverse rupture faults.

Ground motion inputs on both sides of, and close to, surface fault ruptures are critical in determining the reliability of seismic analysis results for fault-crossing bridges. Ground motions on either side of the surface fault rupture primarily stem from three dominant strategies: field-measured methods, hybrid deterministic-stochastic synthetic technology, and the reconstruct ground motion method^20,21^. A few field-measured records near potential faults are significantly affected by baseline drift, attributed to the tilt of the measuring device during intense shaking^22–24^. While numerous baseline correction techniques can effectively restore realistic strong motion records, ensuring the final target displacements align with GPS-measured coseismic offsets is vital^25,26^. Moreover, the hybrid deterministic-stochastic synthetic technology provides an avenue for generating cross-fault ground motions, merging low-frequency (LF) and high-frequency (HF) components using deterministic and stochastic methods respectively. While the hybrid technology offers reasonable fault-crossing ground motion predictions, its adoption is limited due to the absence of pre-developed program codes and the intricate theories behind the stochastic method and Green's function^27,28^. The reconstructed ground motion method, meanwhile, provides an accessible approach to emulate fault-crossing ground motions by integrating expected fling-step and directivity time histories into standard time histories^20,21,29^. The aforementioned reconstructive approach stands out as the most direct and effective way to meet the fault-crossing ground motion requirements for analyzing the seismic behavior of bridges over rupture faults. Thus, we employ this reconstructive approach for our numerical study on the seismic responses of suspension bridges crossing rupture faults^10^.

Research primarily focusing on the seismic responses of simply-supported beam bridges^30^, arch bridges^31^, and cable-stayed bridges^32–35^ has examined bridges crossing active faults using theoretical analysis methods, shaking table tests, and finite element methods. In the realm of theoretical analysis for fault-crossing bridges, Rakesh Goel and Chopra et al.^16,17^ pioneered two practical methods: the response spectrum analysis (RSA) approach and a linear static analysis technique. These were devised to determine the peak response of linearly elastic "ordinary" bridges crossing fault-rupture zones by combining the peak values of quasi-static and dynamic responses. Subsequently, they introduced three approximative methods: modal pushover analysis (MPA), linear dynamic analysis, and linear static analysis, to factor in the nonlinear effects exhibited by ordinary bridges spanning fault-rupture zones. Although the methods introduced by Goel and Chopra have been validated as sufficiently accurate for practical applications^18,19,36^, they come with stringent limitations, including a cap on nonlinearity in the pseudo-static component and a restriction on the span lengths of standard concrete bridges to under 90 m. Regarding long-span suspension bridges, when accounting for geometric nonlinearity and the P-△ effect, the suitability of these streamlined methods warrants further investigation. Pertaining to shaking table tests on fault-crossing bridges, David et al.^37^, over two decades ago, examined the configurations and positions of surface ruptures likely in alluvium atop active dip-slip faults. They carried out tests using both dense and loose sand inside a glass-walled fault testing chamber. Ultimately, they proposed a straightforward model to predict the configurations and positions of surface ruptures, elucidating the functional relationship among the rupture shapes, soil depth, soil's dilation angle, and the fault's dip angle. This study provided an early in-depth analysis of ground surface ruptures arising from potential active faults. However, it didn't encompass bridge structures or consider the uneven distribution of the soil. Saiidi et al.^38^ executed a shaking table test on a large-scale, two-span, reinforced concrete bridge, methodically examining the impact of fault ruptures on the seismic behavior of bridge systems traversing active faults. The findings revealed that ground surface ruptures significantly influenced the nature and position of damages in the bridge bents. Notably, the more flexible bent proximate to the fault was likely to incur the most severe damage. Drawing from a shaking table test on a 1/10 scale model of a two-span, simply-supported bridge, isolated using lead rubber bearings, Jiang et al.^39^ delved into the reactions of isolated, simply-supported bridges experiencing fault dislocation. Observations indicated that employing the lead rubber bearing effectively reduced damage to the girders and piers of the isolated bridge when facing fault ruptures. However, this came with significant displacement responses and either residual deformation or functional impairment of the lead rubber bearing. Xiang et al.^40^ examined the impact of lead rubber bearings and two types of transverse restraining systems—one with shear keys and one without—on the seismic behavior of a bridge isolated from an active fault. The conclusions drawn were consistent: piers distanced from the surface rupture trace are more vulnerable than those nearby. Additionally, shear keys proved notably effective in controlling bearing displacement under conditions of significant permanent surface rupture offsets. Building on both experimental and numerical analyses, Lin et al.^14,41,42^ evaluated the seismic resilience of a steel–concrete composite rigid-frame bridge facing earthquake-triggered ground dislocation at a thrust fault. They systematically analyzed key parameters, including fault location, fault-crossing angle, and fling-step. Additionally, the proposed steel–concrete composite rigid-frame bridge demonstrated considerable seismic resistance capability. Beyond the theoretical analysis and shaking table tests, the sophisticated finite element method is frequently employed to examine the seismic behavior of bridges traversing active faults. Utilizing a multi-degree of freedom model, Park et al.^43^ assessed the Bolu Viaduct's seismic resilience. This viaduct was equipped with a seismic isolation system, including yielding-steel energy dissipation units and sliding pot bearings, and it experienced a surface rupture during the 1999 Turkey earthquake. Numerical assessments of the Bolu Viaduct considered the impact of both the directivity pulse aligned with the fault's normal and the fling step in its parallel direction. The findings indicated that, early in the earthquake, the relative displacement between the superstructure and the pier surpassed the bearings' capacity, subsequently causing damage to both the bearings and the energy dissipation units. Moreover, the study revealed that shear keys, positioned both longitudinally and transversely, played a pivotal role in mitigating damage and preventing potential deck span collapses. Ucak et al.^30^ devised a spectrum of strong ground motions specifically for the finite element evaluation of the Bolu Viaduct, incorporating the influences of the fault rupture zone. They also analyzed a proposed retrofit seismic isolation system for the Bolu Viaduct, juxtaposing it with the initial design methodology. This study provided an in-depth insight into the response of seismically isolated bridges traversed by fault rupture zones. Yang et al.^44^ highlighted the role of permanent ground displacement in determining the seismic behavior of seismically isolated bridges either closely adjacent to or directly traversing a surface fault rupture. Clearly, the impact of permanent ground displacement on the dynamic responses of large-scale engineering structures intersecting fault rupture regions is paramount in the seismic design of bridges spanning these faults. Ge and Saiidi et al.^45^ explored the effects of near-fault ground motions—specifically, a fault-normal directivity pulse and a fault-parallel fling step pulse—on the behavior of a standard three-span reinforced concrete bridge. This bridge integrated Nickel-Titanium (N_i_T_i_) SMA reinforcement at its plastic hinge region and utilized ECC throughout its column. Recently, Zeng et al.^46^ introduced a multi-criteria optimization method to examine the damping impact of an enhanced damper system in a cable-stayed bridge spanning a strike-slip fault. The study systematically assessed how varying fault-crossing angles and earthquake characteristics, including permanent displacements and the ratio of maximum to permanent displacement, influence the damping performance of the optimized damper system. Lin et al.^41^ investigated the seismic behavior and potential damage reduction of a steel–concrete composite rigid-frame bridge under fault-crossing ground motions, utilizing the explicit dynamic analysis platform LS-DYNA. This study offers valuable insights for the seismic design of analogous bridges. Zhang et al.^9^ examined the dynamic behavior of fault-crossing, simply-supported highway bridges. They took into account the influence of the fling-step effect in the fault-parallel direction, the forward directivity effect in the fault-normal direction, fault-crossing angles ranging from 15° to 165°, and the amplitude of permanent ground rupture displacement. The study suggested that the fault-crossing angle and permanent surface rupture offset were pivotal factors influencing the responses of fault-crossing bridges. Concurrently, Zhang et al.^13^ introduced a seismic cable restrainer design method to manage the extensive displacement in multi-span, simply-supported bridges over faults. They compared two restrainer types: elastic steel and superelastic shape memory alloy (SMA) cables. Their findings indicated that using SMA cables as seismic restrainers could significantly decrease the required length in comparison to elastic steel cables. Yang et al.^10^ carried out an extensive parametric study on both ordinary and seismically isolated bridges crossing strike-slip faults using nonlinear time history analysis. Their research evaluated the impact of several factors, including fault crossing angle, location, pier height, span length, seismic excitation polarity, and the number of ground-motion components on the bridge responses. The relative significance of the studied parameters was determined to offer guidelines for the seismic design of bridges crossing potentially active faults. Jia et al.^47–49^ conducted a study on the interaction between the rail tracks and bridges of cross-fault suspension bridges, as well as a dynamic response analysis of suspension and cable-stayed bridges under the impact of pulse-like earthquakes. Despite the evident appeal of long-span suspension bridges with robust spanning capabilities for crossing potential active faults, as highlighted in the aforementioned discussions, there is a noticeable lack of research specifically on fault-crossing suspension bridges. Comprehensive studies on fault-crossing suspension bridges are imperative to establish clear specifications and guidelines for designing long-span suspension bridges in the immediate vicinity of potential active faults.

**Figure 1:**
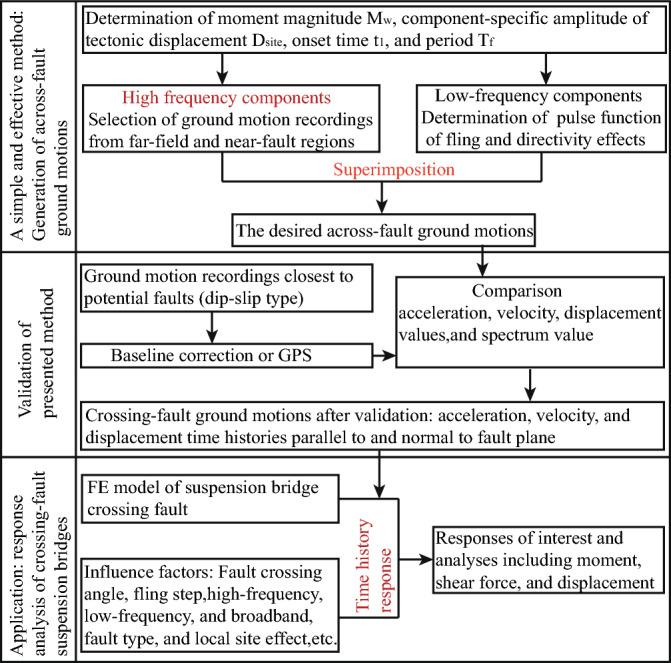
Flowchart of generation of across-fault ground motions and its application.

To provide in-depth insights to the dynamic behavior of long-span suspension bridges subjected to the across dip-slip fault ground motions, this study presents a simple method (Namely adding the LF component of fling step and directivity effects to processed ground motion time history represented as the HF component) to predict and generate the desired and available ground motions in the close proximity of fault-rupture plane for the requirement of seismic analysis and seismic design of various long-span bridges in engineering application field, especially the long-span flexible suspension bridges. The accuracy and effectiveness of the presented method are confirmed by comparing the synthetically generated ground motions with the actual measured ones. A detailed parametric analysis, considering aspects such as fault-crossing location, fault-crossing angle, and frequency components of ground motions at rupture fault sites, is executed using the renowned ANSYS software platform. While the rupture directivity effect is acknowledged as influencing the characteristics of near-fault ground motions, it doesn't receive particular emphasis in this study due to our primary focus on the fling-step effect. The analytical flowchart detailing the generation of dip-slip fault ground motions and the dynamic responses of long-span suspension bridges across active faults is presented in Fig. 1.

## Generation of ground motions on both sides of a dip-slip fault

In reality, recording ground motions directly at the side edges of a fault plane is highly improbable since seismic devices are seldom positioned precisely on these edges of the fault rupture trace. The position of the fault rupture trace during earthquakes exhibits significant variability, influenced by factors like magnitude, attenuation characteristics, fault type, overburden thickness, and structural weight, among others. However, it is feasible to record ground motions that are relatively close to the fault rupture trace. Consequently, these recordings from positions near the fault plane can be used to reconstruct the necessary across-fault ground motions. To address the challenges arising from the lack of measured across-fault ground motions for the seismic design of fault-crossing bridges, we introduce a simple yet effective method to predict and generate ground motions near fault-rupture planes. Additionally, we leverage measured near-fault ground motions from the PEER database, which are as proximate to the fault as feasible, to reconstruct ground motions on the fault plane's side edges, meeting the seismic analysis requirements for fault-crossing bridges. Initially, we conduct parameterization for the fling step parallel to, and the directivity normal to, the fault plane. Subsequently, LF components of the fling-step and directivity pulses are integrated into the processed ground motions, deemed as HF components. Ultimately, the newly generated ground motions, serving as seismic inputs for bridges spanning faults, are validated through comparison with recorded ground motions.

### LF components modelling of fling-step and rupture directivity effects

As is widely acknowledged, numerous scholars have proposed pulse functions characterizing the fling-slip effect and the rupture directivity effect of near-fault ground motions. This study employs the Kamai model^20^, noted for its simplicity of form and clarity of concept. It's recognized that the destructive pulse effect in near-fault ground motions arises from the fling-step effect, parallel to the fault plane, and the directivity effect, perpendicular to it. The introduced method will parametrically represent the pulse effects stemming from both the fling-step and directivity effects. Thus the single cycle of a sine wave is utilized to parameterize the fling step and the acceleration ${a}_{{\text{f}}}\left(t\right)$, velocity ${v}_{{\text{f}}}(t)$, and displacement ${d}_{{\text{f}}}(t)$ time histories forms are given as follows^20^

1 $$ a_{{\text{f}}} \left( t \right) = \left\{ {\begin{array}{*{20}l}  {0 } & \;\;\; { t \le t_{1} }  \\ {\frac{{2\pi D_{{{\text{site}}}} }}{{T_{{\text{f}}}^{2} }}{\text{sin}}\left[ {\frac{2\pi }{{T_{{\text{f}}} }}\left( {t - t_{1} } \right)} \right]} & \;\;\; { t_{1} \le t < t_{1} + T_{{\text{f}}} }  \\ 0 & \;\;\; { t \ge t_{1} + T_{{\text{f}}} } \\ \end{array} } \right. $$

2 $${v}_{f}(t)=\left\{\begin{array}{l}\begin{array}{cc}0 & t\le {t}_{1}\end{array}\\ \begin{array}{cc}\frac{{D}_{{\text{site}}}}{{{\text{T}}}_{{\text{f}}}}-\frac{{D}_{{\text{site}}}}{{{\text{T}}}_{{\text{f}}}}{\text{cos}}[\frac{2\pi }{{T}_{{\text{f}}}}(t-{t}_{1})]& {t}_{1}\le t<{t}_{1}+{T}_{{\text{f}}}\end{array}\\ \begin{array}{cc}0& t\ge {t}_{1}+{T}_{{\text{f}}}\end{array}\end{array}\right.$$

3 $${d}_{f}(t)=\left\{\begin{array}{l}\begin{array}{ll}0 & t\le {t}_{1}\end{array}\\ \begin{array}{cc}\frac{{D}_{{\text{site}}}}{{{\text{T}}}_{{\text{f}}}}(t-{t}_{1})-\frac{{D}_{{\text{site}}}}{2\pi }{\text{sin}}[\frac{2\pi }{{T}_{{\text{f}}}}(t-{t}_{1})]& {t}_{1}\le t<{t}_{1}+{T}_{{\text{f}}}\end{array}\\ \begin{array}{cc}{D}_{{\text{site}}}& t\ge {t}_{1}+{T}_{{\text{f}}}\end{array}\end{array}\right.$$

in which D_site_ denotes the component-specific amplitude of tectonic displacement obtained from field investigation and is in the units of centimeters, T_f_ is the sine-wave period, and t_1_ is the triggered time of the fling step.

Generally the predicted model of the amplitude of tectonic displacement D_site_ can be determined by following formula

4 $${D}_{site}=\left\{\begin{array}{l}\begin{array}{cc}{10}^{{D}_{{\text{fault}}}[{{\text{log}}}_{10}\left({a}_{0}\right)+{a}_{2}{{\text{log}}}_{10}(\frac{{R}_{{\text{rup}}}+{a}_{3}}{{a}_{3}})]}& {R}_{x}<0 \quad {\text{Footwall}}\end{array}\\ \begin{array}{cc}{10}^{{D}_{{\text{fault}}}[{{\text{log}}}_{10}\left({a}_{0}+{a}_{4}\right)+{a}_{2}{{\text{log}}}_{10}(\frac{{R}_{{\text{rup}}}+{a}_{3}}{{a}_{3}})]}& {R}_{x}>0 \quad \mathrm{Hanging wall }\end{array}\end{array}\right.$$

where *D*_fault_ is the average value of slip over the rupture plane and it is a function of moment magnitude M_w_, namely ln(*D*_fault_) = 1.15M_w_-3.28. This model of Eq. (4) expression which has been validated against a set of 84 empirical seismic records with fling-step effect can effectively overcome the defects of ground motion overestimation from the model proposed by Byerly and DeNoyer^50^ and followed by Abrahamson^21^ through increasing the sample number of events and recordings to contain the distance attenuation of fling-step amplitude based on the extensive finite-fault simulations. For various fault types such as dip-slip reverse fault the parameters ${a}_{0}$,${a}_{1}$,${a}_{2}$, and ${a}_{4}$ are listed in Table 1.

**Table 1:** Parameters of the D_site_ model for reverse faults^20^.

Fault type	Reverse fault
Horizontal component	${a}_{1}=f\left({M}_{{\text{w}}},\delta \right)=0.0256{M}_{{\text{w}}}+0.33{\text{ln}}\left(\delta \right)-1.02$ ${a}_{2}=f\left({M}_{{\text{w}}}\right)=19.32{M}_{{\text{w}}}-1.21{M}_{{\text{w}}}^{2}-79.95$ ${a}_{3}=50$ ${a}_{4}=f\left({M}_{{\text{w}}},\delta \right)=3.95-0.98{\text{ln}}(\delta )-0.045{M}_{{\text{w}}}$
Vertical component	${a}_{0}=f\left(\delta \right)=0.0063\delta -0.17$ ${a}_{1}=f\left({M}_{{\text{w}}},\delta \right)=-11.31{M}_{{\text{w}}}^{2}+\left(176.7-0.158 \delta \right){M}_{{\text{w}}}+1.218 \delta -700$ ${a}_{3}=$ 100 ${a}_{4}=f\left({M}_{{\text{w}}},\delta \right)=-0.003\delta +1.555-0.11{M}_{{\text{w}}}$

For the period *T*_f_ of fling-step pulse related significantly to the style of faulting and magnitude two predicting models were developed separately for strike-slip and reverse mechanism such as ${\text{ln}}\left({T}_{{\text{f}},{\text{ss}}}\right)=1.16{M}_{{\text{w}}}-6.42$ (Strike slip fault) and ${\text{ln}}\left({T}_{{\text{f}},{\text{Rev}}}\right)=0.7{M}_{{\text{w}}}-3.54$ (Reverse fault)^20^. Because the fling-step period *T*_f_ in an earthquake event comes from the same fling-step pulse effect it would be considered to be the fling-step period *T*_f_ in the horizontal and vertical directions are the same. Apart from the period *T*_f_ of fling-step pulse the onset time *t*_1_ of the fling-step pulse is another important parameter in the reconstruction process of ground motions on the two sides of a fault plane. Mavroeidis and Papageorgiou^29^ found that the arrival time of the long-period pulse from the fling-step effect was in agreement with the arrival time of the seismic energy radiated from the high velocity region of the rupture. Meanwhile it is observed that this phenomenon are also identical with the observation from the real measured time histories of ground motions^21,51^. Hence, the arrival time of the fling-step effect is approximately considered to align with the arrival of the primary S wave pulse. In this study, we employ a precise method proposed by He et al.^52^ to ascertain the S wave's arrival time through two steps: 1. Determining the preliminary approximate arrival time using the spatial energy gradient curve of three-component ground motions and the short time average (STA)/ Long time average (LTA) method, and 2. Defining the exact arrival time through the quadratic auto-regressive model.

Similarly the directivity pulse is also expressed using simple trigonometric function and the acceleration ${a}_{{\text{d}}}\left(t\right)$, velocity ${v}_{{\text{d}}}(t)$, and displacement ${d}_{{\text{d}}}(t)$ time histories forms are obtained as follows^43,53^

5 $${a}_{{\text{d}}}\left(t\right)=\left\{\begin{array}{l}\begin{array}{ll}0&\quad t\le {t}_{1}\end{array}\\ \begin{array}{ll}\frac{2\pi {V}_{{\text{d}}}}{T}{\text{cos}}\left(\frac{2\pi }{T}t\right)&\quad {t}_{1}\le t<{t}_{1}+{T}_{{\text{d}}}\end{array}\\ \begin{array}{ll}0&\quad t\ge {t}_{1}+{T}_{{\text{d}}}\end{array}\end{array}\right.$$

6 $${v}_{{\text{d}}}\left(t\right)=\left\{\begin{array}{l}\begin{array}{ll}0&\quad t\le {t}_{1}\end{array}\\ \begin{array}{ll}{V}_{{\text{d}}}{\text{sin}}\left(\frac{2\pi }{T}t\right)&\quad {t}_{1}\le t<{t}_{1}+{T}_{{\text{d}}}\end{array}\\ \begin{array}{ll}0&\quad t\ge {t}_{1}+{T}_{{\text{d}}}\end{array}\end{array}\right.$$

7 $${d}_{{\text{d}}}\left(t\right)=\left\{\begin{array}{l}\begin{array}{ll}0&\quad t\le {t}_{1}\end{array}\\ \begin{array}{ll}\frac{T{V}_{{\text{d}}}}{2\pi }\left[1-{\text{cos}}\left(\frac{2\pi }{T}t\right)\right]&\quad {t}_{1}\le t<{t}_{1}+{T}_{{\text{d}}}\end{array}\\ \begin{array}{ll}0&\quad t\ge {t}_{1}+{T}_{{\text{d}}}\end{array}\end{array}\right.$$

where *V*_d_ and T_d_ imply the peak ground velocity and period of the directivity pulse. For a special earthquake with *M*_w_, *V*_d_ can be calculated according to the estimation by Somerville^54^. Also the period T_d_ will be obtained thanks to the following relationship of the period of the directivity pulse and the earthquake magnitude^54^.

8 $$ T_{{\text{d}}} = 10^{{0.36M_{{\text{w}}} - 2.02}} \quad {\text{For}}\,{\text{soil}}\,{\text{sites}} $$

Despite the square pulse function were also proposed by other scholars to represent the pulses of near-fault ground motions the trigonometric function used herein is better to describe the abrupt acceleration discontinuities and provide a simple and closed-form formulations of acceleration, velocity, and displacement histories.

### HF components of fault-crossing ground motions

Beyond the LF components stemming from the fling-step and directivity pulses, the HF components of fault-crossing ground motions play a crucial role in influencing the responses of fault-crossing bridges. Recent major earthquakes, such as the Loma Prieta event (17 October 1989), the Chi-Chi quake (21 September 1999), and the Wenchuan disaster (20 May 2008), showed minimal attenuation in the propagation of HF ground motion components from the epicenter to sites in the near-fault region^9,20,21,50^. Therefore, the HF components in the near-fault region can be viewed as analogous to the corresponding components of fault-crossing ground motions, especially when actual measurements from large-magnitude, close-proximity seismic events are lacking. This means that determining the HF components of fault-crossing ground motions essentially becomes a matter of selecting the appropriate ground motions from near-fault regions. Typically, one can obtain the desired near-fault ground motions by matching them to a target response spectrum, based on factors such as fortification intensity, seismic group, and site soil classification. At times, one can directly choose near-fault ground motions that match in terms of seismic source type, magnitude, site soil type, and proximity to the epicenter, among other factors. To best meet the needs of seismic analysis for specific sites, one can adjust or appropriately scale the peak acceleration (PGA) of the chosen ground motions, building upon the aforementioned approach.

**Figure 2:**
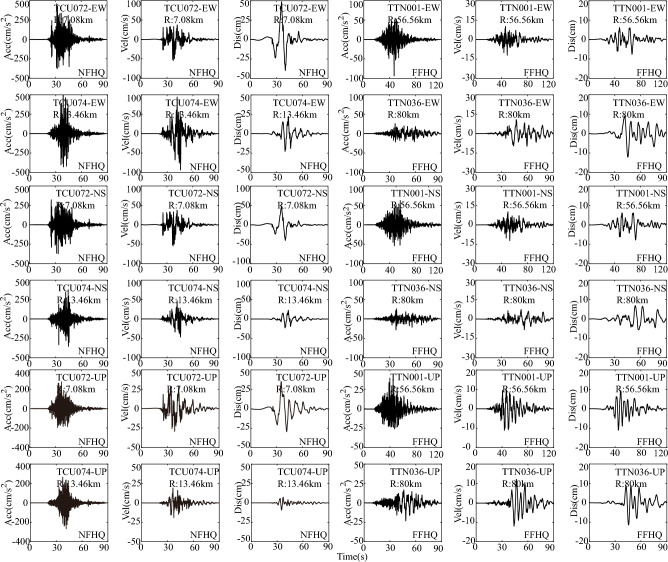
High frequency components of examined ground motions from the Chi-Chi earthquake in 1999 (Dip-slip fault). Note: Acc: acceleration time history; Vel: velocity time history; Dis: displacement time history; NFHQ: near-fault HF component; FFHQ: far-field HF component; R: distance from station to rupture fault; TCU072, TCU074, TTN001, and TTN036 represent station name; EW: East–west direction, NS: North–south direction, and Up: vertical direction.

For comparative purposes, both far-field and near-fault ground motions from the PEER database are directly chosen to represent the HF components on either side of a specific rupture fault plane. Far-field ground motions are sourced from the PEER database within a distance range of 30–100 km, signifying that R falls within the 30–100 km interval. Likewise, the near-fault ground motions are characterized by R being 30 km or less. In this study, we will consider the influence of the dip-slip fault on the responses of long-span suspension bridges. Detailed information on ground motions with HF components from a representative dip-slip fault, namely the Chi-Chi earthquake of 1999, is depicted in Fig. 2.

### Generation of ground motions on two edges of a given fault plane

**Figure 3:**
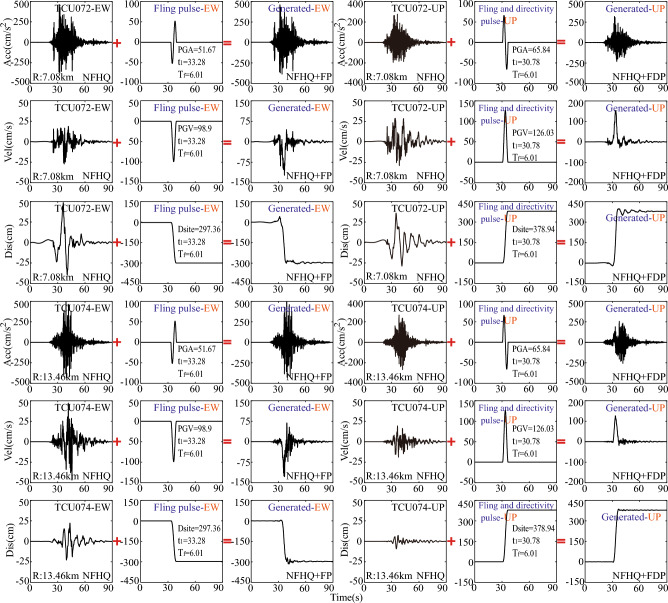
Generated dip-slip ground motions based on the HF components from near-fault region (TCU072 and TCU074 stations) in Chi-Chi earthquake. Note: FP-Fling pulse and FDP-Fling and directivity pulse.

In this section, new ground motions generated across a dip-slip fault will be obtained by adding a predicted surface rupture displacement induced by faulting to the processed ground motion time histories (HF components). Given the limited space only the artificially generated hanging wall ground motions are displayed herein in the Fig. 3. For dip-slip fault scenarios, HF components were extracted from time history recordings at four stations (e.g., TCU072, TCU074, TTN001, and TTN036) using a high-pass filter with a frequency cutoff of 0.2 Hz, effectively eliminating LF components. Both HF and LF components from the east–west (EW) and up-down (UP) directions are considered here, as opposed to the north–south (NS) direction, due to the idealized nature of the dip-slip fault model. To simplify, only one process of reconstructing the across-fault ground motion based on the HF components of the time history recordings from TCU072 station is detailed. Initially, the time history recordings from the TCU072 station in the EW direction are chosen from the PEER database, followed by the utilization of a high-pass filter with a frequency of 0.2 Hz to remove the interference of other LF noise components. The amplitude of tectonic displacement of D_site_ = 297.36(cm) can be predicted by the Eq. (4) in the condition of M_w_ = 7.6 and δ = 30° in Chi-Chi earthquake. The onset time of t_1_ = 33.28(s) and the fling step period of T_f_ = 6.01(s) are obtained by He’s method^50^ and the model of ${\text{ln}}\left({T}_{{\text{f}},{\text{Rev}}}\right)=0.7{M}_{{\text{w}}}-3.54$. Finally, the predicted LF fling pulse is spliced or added to the HF components in the near-fault regions to generate the across-fault time history of ground motion shown in Fig. 5a. Meanwhile in the UP direction the across-fault time history of ground motion in the Fig. 3 is obtained in accordance with these parameters of D_site_ = 130.4 (cm), t_1_ = 33.78(s), and T_f_ = 6.01(s). As prominently demonstrated in Fig. 3, the characteristics of the across-fault time histories of ground motions in both the EW and UP directions, as shown in Fig. 3, are restored, including the ground rupture permanent displacements originating from the fling-step and directivity pulses.

**Figure 4:**
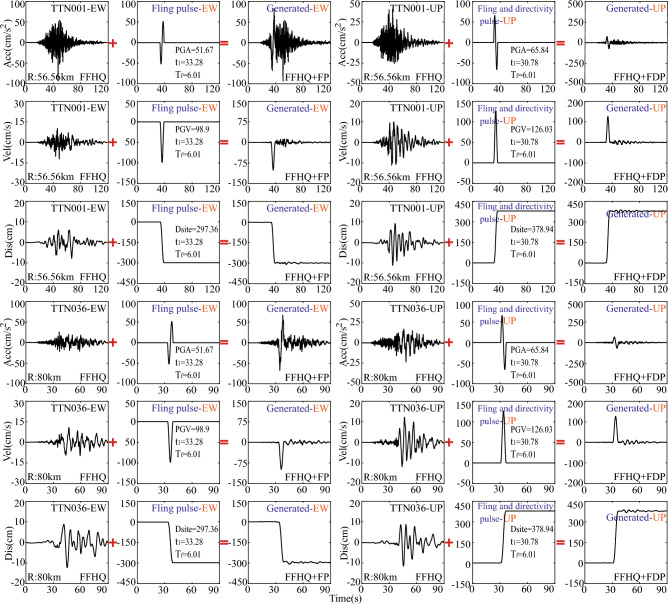
Generated dip-slip ground motions based on the HF components from far-field region (TTN001 and TTN036 stations) in Chi-Chi earthquake.

To compare the across-fault ground motions reconstructed based on near-fault ground motions in both the east–west (EW) and up-down (UP) directions, the corresponding generated across-fault ground motions in Fig. 4 are also provided, relying on far-field ground motions from TTN001 and TTN036 stations. Regardless of whether the HF components of reconstructed across-fault ground motions originate from near-fault regions or far-field areas, the simple method presented in this study can effectively produce the desired across-fault ground motions with ground rupture permanent dislocation. In other words, this method can readily obtain across-fault ground motions related to dip-slip faults at various earthquake intensity levels.

### Validation and analysis of generated across fault ground motions

**Figure 5:**
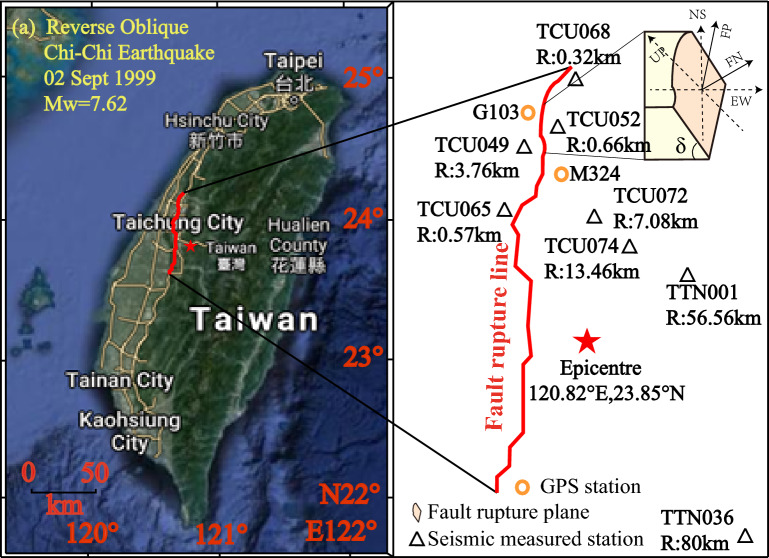
Arrangement of seismic array and GPS station in Chi-Chi earthquake.

Prior to employing reconstructed across-fault ground motions for seismic analysis, validating their alignment with empirical observations is imperative. For validation purposes, this study utilizes the Chi-Chi earthquake as a representative model of dip-slip fault behavior to assess the generated across-fault ground motions. This selection is predicated on the extensive availability of seismic and geodetic array GPS stations, offering recordings and data crucial for accurately estimating coseismic displacements, as depicted in Fig. 5. The Taiwan Chi-Chi earthquake, which struck on September 21, 1999, with a moment magnitude of 7.6, caused a surface rupture of approximately 100 km, predominantly along the established north–south Chelungpu fault. This fault is classified as a reverse oblique dip-slip fault. Situated just 0.66 km from the fault plane, the TCU052 station is the nearest station to the Chi-Chi earthquake's epicenter. Consequently, after baseline correction using data from the nearest GPS station, the recordings at TCU052 station are deemed representative of actual across-dip-slip fault ground motions. For validation, the reconstructed across-fault ground motions will be compared with TCU052 station recordings, to assess the accuracy of corrections applied to artificially generated across-dip-slip fault motions. The detailed validation scheme procedure is provided as follows.

1. Choosing HF components from far-field earthquakes (Seismic array stations: TTN036 and TTN001) and near-fault earthquakes (Seismic array stations: TCU074 and TCU072);
2. Identifying LF pulse components, including the fling step pulse parallel to the fault plane and the directivity pulse normal to the fault plane;
3. Integrating the pulse components into the processed ground motions to create synthetic ground motions across the fault;
4. Contrasting synthetic ground motions across the fault with time history recordings from TCU052 station during the Chi-Chi earthquake, examining both time and frequency domains.

**Figure 6:**
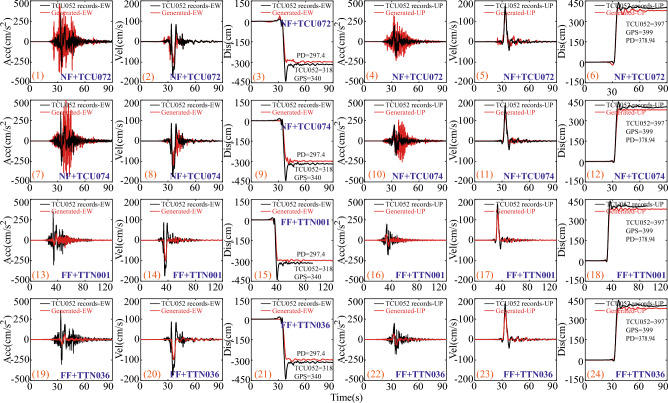
Comparison of generated across fault ground motions and TCU052 recordings in 1999 Chi-Chi earthquake.

Furthermore, a detailed comparison between the reconstructed ground motions associated with dip-slip faults and the actual ground motion records from TCU052 station is presented in Fig. 6. In the east–west (EW) direction, the time histories of acceleration, velocity, and displacement for the simulated across-fault ground motions, derived from the HF components of TCU072 and TCU074 stations in near-fault regions, are juxtaposed with the ground motion records at TCU052 station, as depicted in Fig. 6 (1)–(3) and (7)–(9). As is readily evident, the components in the east–west (EW) direction align closely with the TCU052 records. Furthermore, the permanent surface rupture displacements in Fig. 6 (3), (9), (15), and (21) measure 318 cm and 340 cm, recorded at TCU052 station and M324 GPS station, respectively. The corresponding permanent displacement (PD) for the reconstructed across-fault ground motions measures 297.4 cm. Clearly, this value is in substantial agreement with the previous two measurements in the east–west (EW) direction. Undoubtedly, this validates the feasibility and effectiveness of the method proposed in this study. In the up-down (UP) direction, the permanent displacement (PD) value of 397 cm recorded at TCU052 station closely corresponds to the observed value of 399 cm at the M324 GPS station, despite the several-kilometer separation between the two arrays. As illustrated in Fig. 6 (6), (12), (18), and (24), the predicted PD value of 378.94 cm, situated at the side edge of the dip-slip fault plane, is in substantial agreement with the observations. It is quite evident that there is a noticeable deviation between the predicted PD and the PDs recorded at TCU052 and M324 GPS stations, as can be observed in Fig. 6. However, the general trend shows remarkable consistency between the predicted PD and the PDs recorded at TCU052 and M324 GPS stations. The primary factors contributing to the deviation are as follows: (1) The nature of D_fault_ in Eq. (4), representing the average slip over the rupture plane, can result in deviations when predicting the PD value at specific points, notably at locations with fault plane discontinuities; (2) The spatial separation spanning several kilometers between assessment points, such as the fault's side edge, TCU052 station, and PGS M324 location, also plays a role in the observed deviation; (3) Discrepancies partially arise from factors like noisy signals, site tilting, sensor orientation errors, among others; (4) Complex source properties, seismograph site conditions, sampling frequencies, and propagation paths introduce uncertainties, thereby contributing to the study's observed deviations. Similarly, time histories of acceleration, velocity, and displacement in the EW and UP directions for simulated across-fault motions, based on HF components from TTN001 and TTN036 in far-fault regions, are contrasted with TCU052 records [Fig. 6 (13) to (24)]. Reconstructing across-fault motions using far-field components shows similar patterns and results as those predicted from HF components of near-field quakes. Disparities between the predicted across-fault motions and array station records are attributable to station separation, fault discontinuities, complex seismic sources, noise sources, and attenuation relations, among others.

**Figure 7:**
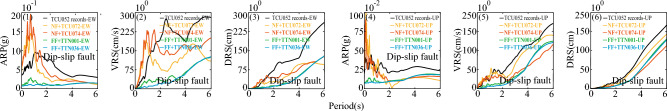
Comparison of generated across fault ground motions and real records from array station in frequency domain.

In addition to the time domain comparison, the ground motion time histories are transformed into response spectra for a frequency domain comparison, as illustrated in Fig. 7. It's evident from Fig. 7 (1) that when compared to the response spectrum derived from the ground motion recordings at TCU052 station, the response spectra from the generated ground motions at TCU072 and TCU074 stations in the near-fault regions contain a more pronounced presence of HF components, typically within the period range of 0-1 s. However, within the period range of 1–6 s, it is the LF components in the ground motion recordings at TCU052 station that are most pronounced. The permanent surface rupture displacement (PD) is largely influenced by these LF ground motion components. This corroborates the findings in Fig. 6 (3) and (9), where the PD (318 cm) in the ground motion recordings at TCU052 station surpasses the PD (297.4 cm) of the generated ground motions at TCU072 and TCU074 stations. It is noteworthy that the response spectra derived from the generated ground motions based on the records from TTN001 and TTN036 stations in far-field regions, as depicted in Fig. 7 (1), exhibit the smallest values in both LF and HF components. This clearly indicates that the ground motions generated from the HF components in near-fault regions better meet the requirements for accuracy compared to those from far-field regions. In other words, the proximity of array stations recording HF components to the fault planes leads to more accurate predictions of across fault ground motions.

## Finite element modelling of the crossing-fault suspension bridge

### Information of the crossing-fault suspension bridge

**Figure 8:**
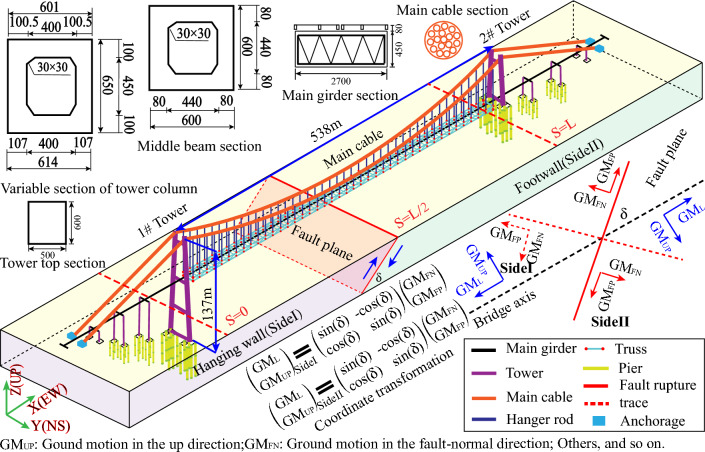
Schematic view of fault seismic ground motions and long-span suspension bridge structure.

In order to investigate the seismic performance of long-span suspension bridges, we focus on a unique long-span suspension bridge situated in the western Guizhou province of China. The employed long-span suspension bridge with layout of 136 m + 538 m + 136 m is composed of main tower, steel truss girder, and main cable and situated on a V-shape gully region with the riverbed length approximate 120 m. The main towers, 137 m in height for 1# tower and 62.5 m for 2# tower, are constructed using a reinforced concrete portal frame structure, supporting a bridge width of 27 m. The detailed information about the long-span suspension bridge is provided in Fig. 8. Furthermore, Fig. 8 illustrates the transformation of ground motions that are both normal and parallel to the faults into ground motions in the X (EW) and Z (UP) directions, facilitating their use as seismic inputs in finite element (FE) analysis. The coordinate transformation equations can be found in Fig. 8.

### Finite element model based on ANSYS software

Utilizing the general finite element platform of ANSYS, a 3D finite element model of the long-span suspension bridge, designed to cross a potential active fault, is developed. The model incorporates Beam189, Mass21, and Shell163 elements from the ANSYS software's element library to represent the main girders and towers, the dead loads applied to the main girders, and the steel deck. The Link10 element, designed to consider only the tensile effect, is utilized for modeling the main cable and hanger rod. It should be noted that the hangers of a suspension bridge only endure tensile axial forces, and their mechanical properties can be accurately reflected by using just a single element for simulation. In addition, the Combin14 element is employed to simulate the bearing and viscous damper installed between the tower and girder. These material parameters (e.g., elastic modulus, Poisson’s ratio, and density) of concrete, steel, and cable are considered as 3.5 × 10^10^, 0.2, and 2887.5 kg/m^3^; 2.1 × 10^11^, 0.3, and 8242.3 kg/m^3^; and 2.0 × 10^11^, 0.17, and 8400 kg/m^3^ respectively. It should be noted that the densities of concrete and steel materials are increased by approximately 10% in a conservative manner (i.e., multiplied by a factor of 1.1) to account for the weight of structural connecting members. The neglect of pile-soil interaction in this analysis is attributed to the firm soil conditions surrounding the piles. The displacement-based seismic excitation mode is employed to address the issue of inputting across-fault ground motions for long-span suspension bridges. This seismic input mode readily accounts for the ground rupture's permanent displacement caused by the fling-step effect and directivity effect. It is deemed reasonable to assume that each segment (located on either side or opposite side of the fault plane) of the long-span suspension bridge divided by the fault plane experiences the same movement. The damping ratio of structure system is assumed to be 0.05. The Rayleigh damping (Mass damping coefficient α = 9.57 × 10^–4^ and stiffness damping coefficient β = 7.879.57 × 10^–2^) in the dynamic analysis is considered herein on the basis of mode frequency of the first 200 orders. In addition, the damping index and the damping coefficient of viscous damper is considered respectively as 0.3 and 5000 kN·s/m after many trial calculations. The two ends of the main girder are treated as having released longitudinal degrees of freedom, as the structural response remains conservative without abutment constraints. The connection between the main cable and tower top is managed using a node-sharing method. The cable and main girder connection releases the torsional degrees of freedom due to the neglect of moment transmission influence in practical engineering applications.

### Cases and ground motion inputs

**Figure 9:**
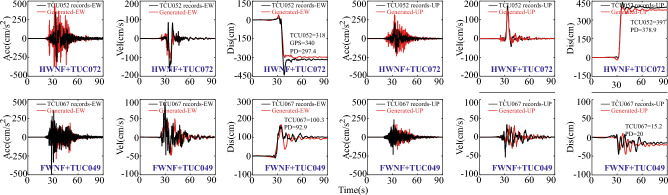
Ground motion inputs for dip-slip faults.

Before conducting this study, it is important to clarify that only 4 artificially generated fault-crossing ground motions are considered due to the difficulty in finding additional measured ground motions to validate the accuracy of the artificial ground motions based on existing literature and collected data^55^. For the purposes of research, three groups, namely Group A (GA), Group B (GB), and Group C (GC), are necessary for this study. Group A is intended to validate the accuracy of the predicted ground motions and to compare the differences between recordings in the array station and predicted ground motion. In the C1 case of Group A, the seismic input in this FE analysis consists of the recordings of TCU052 on the hanging wall and the recordings of TCU067 on the footwall, serving as the measured ground motions (MGM). In addition, for the C2 case in Group A, the corresponding generated ground motions (GGM) based on the HF components of TCU072 station on the hanging wall and TCU049 station on the footwall are prepared for comparative analysis. It is important to note that all ground motion inputs will be scaled to the original amplitude of 0.2 instead of period, as the calculation does not converge due to excessive fault rupture displacements when considering intense geometric nonlinearity in the seismic analysis of long-span suspension bridges. The detailed seismic inputs and case plan can be found in Fig. 9 and Table 2.

**Table 2:** Group A and the parameters of ground motion inputs for dip-slip faults.

Group	Case	Station	Direction	R	PGA (cm/s^2^)	PGV (cm/s)	PGD (cm)	PD (cm)	T_f_ (s)	t_1_ (s)
A	C1	TCU052	EW:(X)	0.66	350.4	174.6	439.5	318	9.6	33.6
			UP: (Z)	0.66	198.1	174.8	438.6	397.0	10.2	31.1
		TCU067	EW:(X)	0.62	491.3	92.0	161.2	100.3	2.3	26.5
			UP: (Z)	0.62	231.3	53.1	45.6	15.2	4.9	29.0
	C2	GGMHW-TCU072	EW:(X)	0.00	456.5	122.9	307.1	297.4	6.0	33.3
			UP: (Z)	0.00	318.6	153.4	398.7	378.9	6.0	30.8
		GGMFW-TCU049	EW:(X)	0.00	287.2	48.9	168.0	92.9	6.0	25.4
			UP: (Z)	0.00	170.0	31.6	45.4	20.0	6.0	27.4

GGMHW-TCU072 represents the generated ground motions on the hanging wall on the basis of the HF components of TCU072 station records; GGMFW-TCU049 indicates the generated ground motions on the footwall on the basis of the HF components of TCU049 station records.

Meanwhile, Group B (GB) aims to investigate the impact of the fault crossing angle (FCA) and the location of the emerging fault rupture trace on the responses of the long-span suspension bridge. Due to the significant uncertainty surrounding the precise fault location and the angle of fault crossing the bridge, this parametric investigation is essential for obtaining response envelopes for the seismic design of bridges crossing faults. This section conducts a parametric study to examine the influence of the various locations of the emerging fault rupture trace relative to the bridge and the fault-crossing angle on the responses of the long-span suspension bridge. Based on the assumption of various positions (S = 0, S = L/2, and S = L; where L represents the main span length and S denotes the relative position to the main tower) of the emerging fault rupture (as shown in Fig. 8), the differences in distance from the fault plane to the bridge tower are simulated using different apparent wave velocities (AWV) propagating on the ground surface, assumed to be 100, 200, 400, 600, and 800 m/s. As the vibration resulting from the fault plane's fracture is transmitted to the bridge tower in the form of highly discrete waves, we employ the comprehensive method. After multiple trial calculations, the AWVs are confined to the range of 100–800 m/s. Beyond 800 m/s, the impact of the location of the emerging fault rupture trace on the responses of the suspension bridge can be disregarded. The angle of fault crossing the bridge is considered from 15° up to 165° with an interval of 15° on each assumed location of the emerging fault rupture. Consequently, the GB will include a series of subgroups such as SGB1, SGB2, and SGB3. Except for SGB2, each subgroup consists of five cases, namely case1 (C1), case2 (C2), case3 (C3), case4 (C4), and case5 (C5). In the GB presented in Table 3, 51 cases will be analyzed to examine the influence of the FCA and the location of the emerging fault rupture trace on the responses of the special long-span suspension bridge.

**Table 3:** The details of Group B.

Group	Subgroup	Location	Case	AWV (m/s)	FCA (°)
B	SGB1	S = 0	C1	100	30, 60, 90, 120, and 150
			C2	200	30, 60, 90, 120, and 150
			C3	400	30, 60, 90, 120, and 150
			C4	600	30, 60, 90, 120, and 150
			C5	800	30, 60, 90, 120, and 150
	SGB2	S = L/2	…	…	30, 60, 90, 120, and 150
	SGB3	S = L	C1	100	30, 60, 90, 120, and 150
			C2	200	30, 60, 90, 120, and 150
			C3	400	30, 60, 90, 120, and 150
			C4	600	30, 60, 90, 120, and 150
			C5	800	30, 60, 90, 120, and 150

Finally, Group C in Table 4 is organized to examine the extent of influence of LF, HF, and broadband frequency (BF) components of across fault ground motions on the responses of the long-span suspension bridge crossing the fault. Group C is divided into five subgroups, namely SGC1, SGC2, and SGC3, to assess the impact of the location of the emerging fault rupture trace on the responses of the long-span suspension bridge. Each subgroup is further subdivided into three cases, C1, C2, and C3, to assess the impact of different frequency components under varying AWVs on the responses of the long-span suspension bridge crossing the fault plane at a 90° angle. It is important to note that the BF ground motions are a combination of LF and HF components. The LF components arise from the projected pulse, while the HF components are derived from HF seismic records. Group C comprehensively examines the impact of frequency components, fault plane location in relation to the main tower, and AWV on the responses of the long-span suspension bridge, encompassing a total of 45 cases.

**Table 4:** The details of Group C.

Group	Subgroup	Location	Frequency	Case	AWV (m/s)	FCA (°)
C	SGC1	S = 0	LF	C1	100, 200, 400, 600, and 800	90
			HF	C2	100, 200, 400, 600, and 800	90
			BF	C3	100, 200, 400, 600, and 800	90
	SGC2	S = L/2	LF	C1	100, 200, 400, 600, and 800	90
			HF	C2	100, 200, 400, 600, and 800	90
			BF	C3	100, 200, 400, 600, and 800	90
	SGC3	S = L	LF	C1	100, 200, 400, 600, and 800	90
			HF	C2	100, 200, 400, 600, and 800	90
			BF	C3	100, 200, 400, 600, and 800	90

## Analysis results and discussion

### Responses comparison of the special crossing-fault bridge

**Figure 10:**
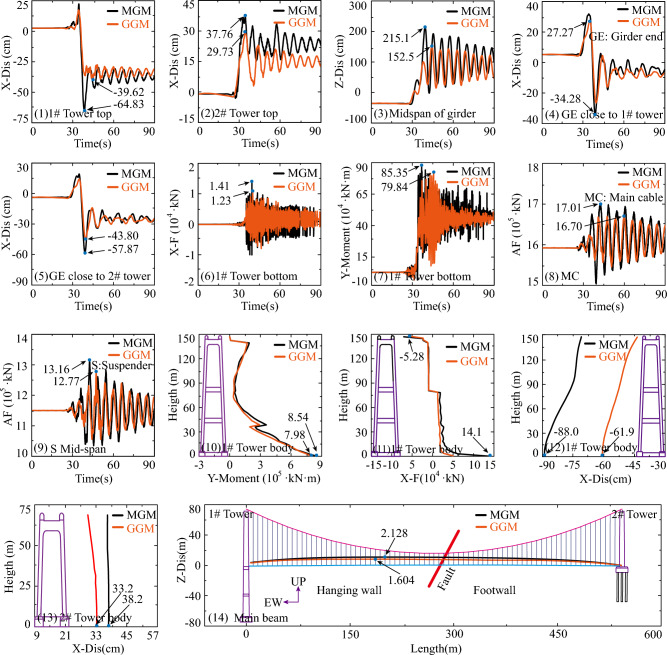
Response results in the Group A.

The results from Fig. 10 indicate a strong correlation between the responses of the special long-span suspension bridge under the MGM and the GGM. Specifically, in Fig. 10 (1) and (2), the displacements of the top of 1# tower and 2# tower in the X direction are analyzed. The maximum displacement at the top of 1# tower, positioned on the hanging wall, is greater than that of 2# tower on the footwall due to the pronounced hanging wall effect. The disparity in tower top displacements and the final permanent rupture displacements of input ground motions on both the hanging wall and footwall is due to the elastic deformation of the main towers. Additionally, in Fig. 10 (3–5), a strong agreement is observed between the MGMs and the GGMs for the vertical displacements (Z direction) at the middle of the main girder and the longitudinal displacements (X direction) close to both 1# tower and 2# tower. The internal force responses of the 1# tower bottom, including X-F (Shear force) in Fig. 10 (6) and the moment (Around Y axial) in Fig. 10 (7) under the MGMs, align with the responses under the GGMs. The disparity between the maximum response values of the 1# tower bottom, including X-F (Shear force) in Fig. 10 (6), and the moment (Around Y axial) in Fig. 10 (7) under the MGMs and the GGMs is 12.8% and 6.5% respectively. As known, the main cable and suspender in the midspan are susceptible to damage during earthquakes. The response comparison of the main cable and the suspender under the MGMs and the GGMs is presented in Fig. 10 (8) and (9). The maximum response difference values of the main cable and suspender in the midspan due to the MGMs and the GGMs are 1.82% and 2.96% respectively. The internal force and displacement responses of the tower, along with the displacement responses of the main girder under the MGMs and the GGMs, are examined and compared in Fig. 10 (10)–(14). Similar consistencies are observed in the comparisons. In summary, it appears that the GGMs meet the application requirements, as the response differences in the seismic analysis of the fault-crossing suspension bridge are small. Therefore, this presented simple and convenient method, which adds the predicted faulting-induced surface rupture displacement of the fling step effect to processed ground motion time histories, can generate the across dip-slip fault ground motions. The accuracy and effectiveness of the presented method are validated in the seismic response analysis of the fault-crossing suspension bridge.

### Effect of fault-crossing angle and the location of emerging fault rupture trace

#### Effect of fault-crossing angle

**Figure 11:**
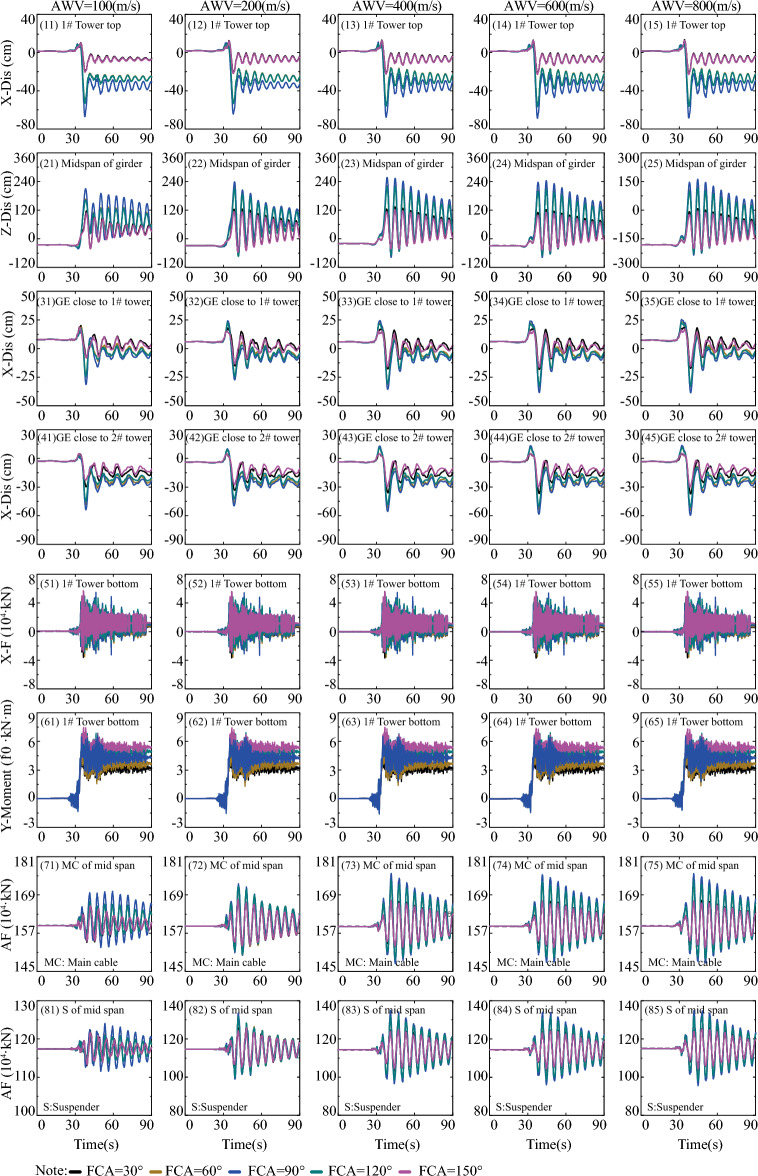
Response results of SGB1(S = 0) in the Group B.

**Figure 12:**
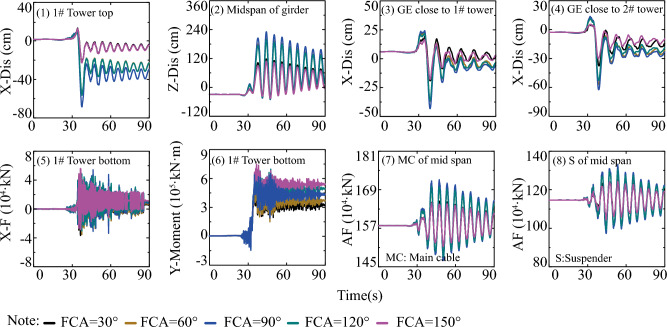
Response results of SGB2(S = L/2) in the Group B.

**Figure 13:**
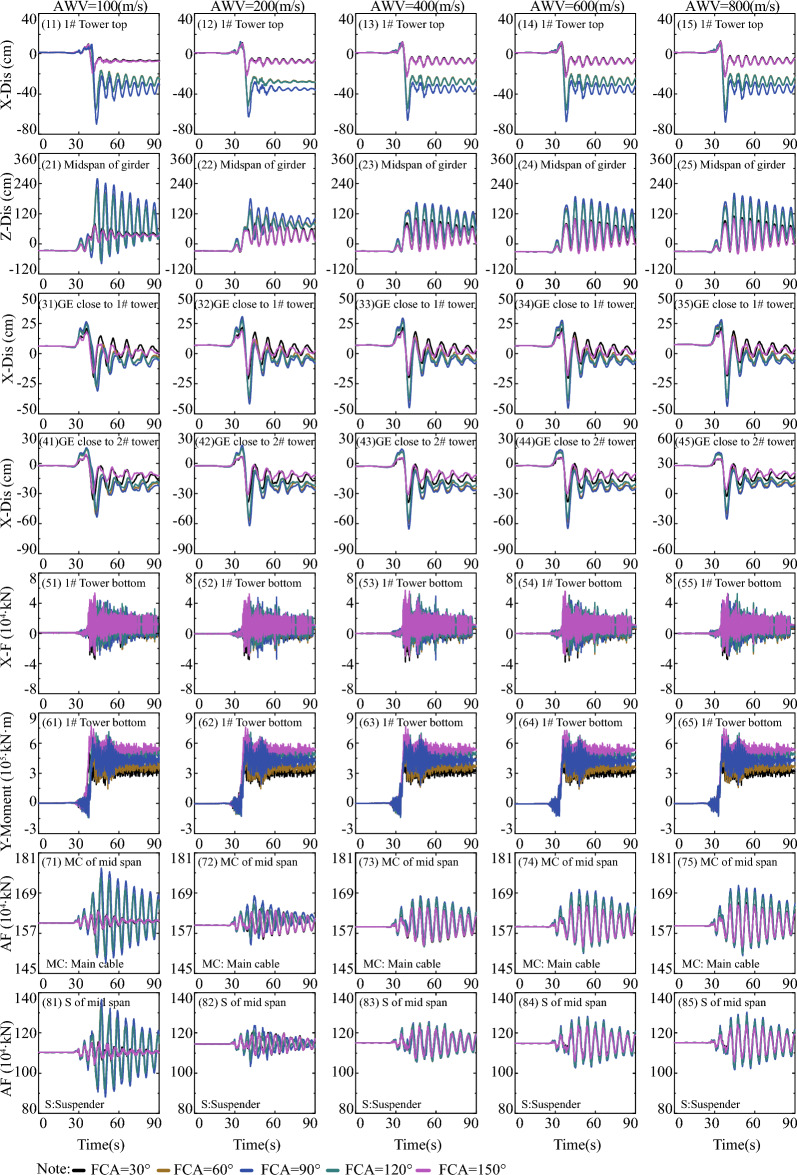
Response results of SGB3(S = L) in the Group B.

To investigate the impact of fault-crossing angles in various fault rupture locations (e.g., S = 0 in Fig. 11, S = L/2 in Fig. 12, and S = L in Fig. 13) on the responses of the specific long-span suspension bridge experiencing faulting, three typical and representative positions (S = 0, S = L/2, and S = L in Fig. 8) where the fault intersects with the bridge are analyzed. A matrix-based graphical representation is employed in Figs. 11 and 13 to enhance reader comprehension. Each column represents the responses of interest with varying FCA at a specific AWV, while each row represents the responses of interest with varying AWV at a specific FCA. As evident from the first four rows in Fig. 11, permanent ground surface rupture displacement plays a crucial role in determining the final displacement requirements of the fault-crossing suspension bridge. Permanent displacements will occur at the 1# tower top, midspan of the girder, GE close to 1# tower, and GE close to 2# tower due to fault dislocation. As depicted in Fig. 11, the displacement and internal force responses are notably the largest when FCA = 90. Perhaps the adoption of 90° as the FCA for the seismic design of long-span suspension bridges across dip-slip faults is not reasonable. Furthermore, from a row perspective, the AWV minimally affects responses of interest, except for the main cable and suspender of the midspan in the 7th and 8th rows in Fig. 11. This suggests that the influence of AWV can be disregarded when the fault trace approaches the 1# tower (S = 0).

**Figure 14:**
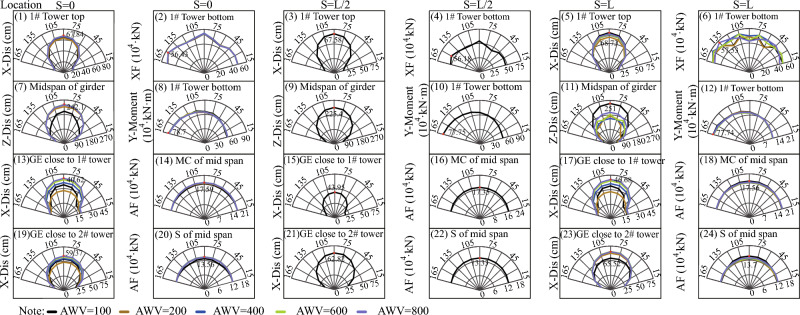
Max peak value of responses with different angles.

Additionally, the response results of SGB2 (S = L/2) are depicted in Fig. 12. When the fault passes through the middle of the main span of the bridge (S = L/2), the arrival times of AWVs from the fault plane to the two ends of the suspension bridge are identical irrespective of AWVs. Thus, it suffices to consider the influence of FCA. The significance of FCA in affecting the responses of the special long-span suspension bridge is apparent in Fig. 12. It is evident from Fig. 12 that the displacement and internal force responses are maximum when FCA = 90°. Generally, the trends observed in Fig. 13 are similar to those in Figs. 11 and 12. Nevertheless, the peak values of vertical displacement responses in the second row of Fig. 13 exhibit an initial decrease followed by an increase with varying AWVs. Furthermore, the response patterns of the main cable and suspender in Fig. 13 differ from those in Fig. 11. This divergence may be attributed to: (1) Varied AWVs resulting in differential seismic wave arrival times at the tower base, leading to asymmetric mode participation; (2) Variances in rupture trace locations between S = 0 and S = L. Similarly, the Fig. 13 illustrates that the displacement and internal force responses are most significant when the FCA = 90°. The maximum peak values of responses, considering different fault-crossing angles (15° to 165°) and locations of fault rupture traces (S = 0, S = L/2, and S = L), are analyzed in Fig. 14. The responses exhibit a notable degree of consistency across different AWVs and demonstrate symmetry about the FCA = 90° axis. With the exception of the responses depicted in Fig. 14 (8), (10), and (12), the majority of key response components reach their maximum values when FCA = 90°. Notably, when the fault rupture trace is in proximity to 2# tower (S = L), the axial force at the base of 1# tower does not reach its maximum at an FCA of 90°, as depicted in Fig. 14 (6). Observing the asymmetrical outcomes around the FCA = 90° axis depicted in Fig. 14 (8), (10), and (12), it can be noted that the pulse direction of the fling-step effect, in conjunction with the counter-clockwise rotation (ranging from 15° to 165°) of the fault plane (as shown in Fig. 8), moves from the positive direction of the X(EW) axis to the opposite direction. Therefore, when analyzing the effects of the fault-crossing angle, the pulse direction of the fling-step effect contributes to increased responses and the observed asymmetry.

#### Effect of fault-crossing location

**Figure 15:**
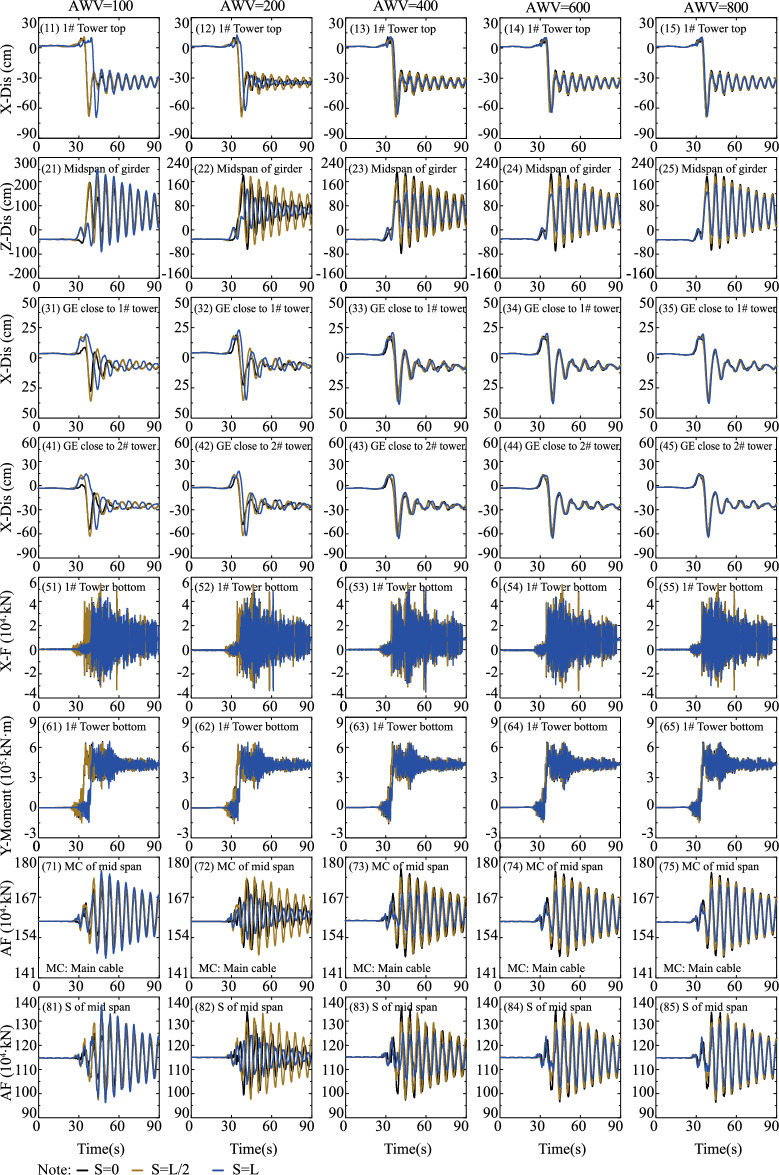
Response results on fault-crossing location and FCA of 90°

This section systematically analyzes the impact of fault-crossing locations on the responses of the long-span suspension bridge subjected to faulting. The first row of Fig. 15 indicates that the position of fault rupture lines minimally affects the displacement of the tower top along the bridge axis (X direction). Additionally, for the vertical displacement of the midspan of the girder (Z-Dis in the second row of Fig. 15), the smallest peak value response occurs when AWV = 200 m/s and S = L. The third and fourth rows of Fig. 15 demonstrate that the impact of the position of fault rupture lines on the responses of GE close to 1# and 2# towers becomes uncertain with the increase of AWVs. Similarly, from the perspective of the internal force response of the main tower in the fifth and sixth rows of Fig. 15, the influence of fault-crossing location is similarly vague, except for a slight time delay under low AWVs. Additionally, for the main cable and suspender, the axial force responses (AF in the seventh and eighth rows of Fig. 15) are minimal when AWV = 200 m/s and S = L. The negligible difference in the seismic design of fault-crossing suspension bridges can be attributed to the asymmetry of the suspension bridge structure and time-lag effect.

**Figure 16:**
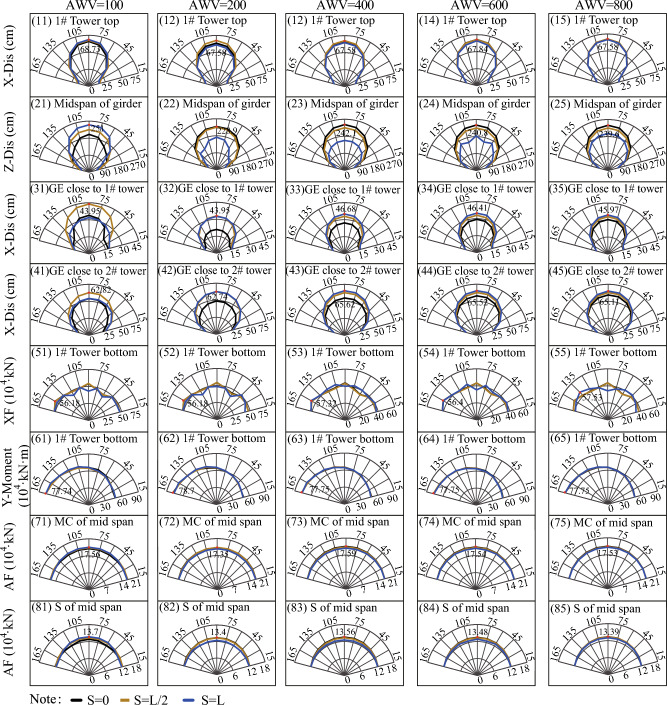
Max peak values with various FCAs and fault rupture positions.

The Fig. 16 illustrates the maximum peak values of responses with various FCAs. Apart from the displacement responses of the girder in the second, third, and fourth rows of Fig. 16, the response curves of other parameters almost entirely overlap. This observation suggests that the influence of fault rupture position on the girder responses can be disregarded as the AWVs increase.

#### Combination effect of fault-crossing angle, location, and AWV

**Figure 17:**
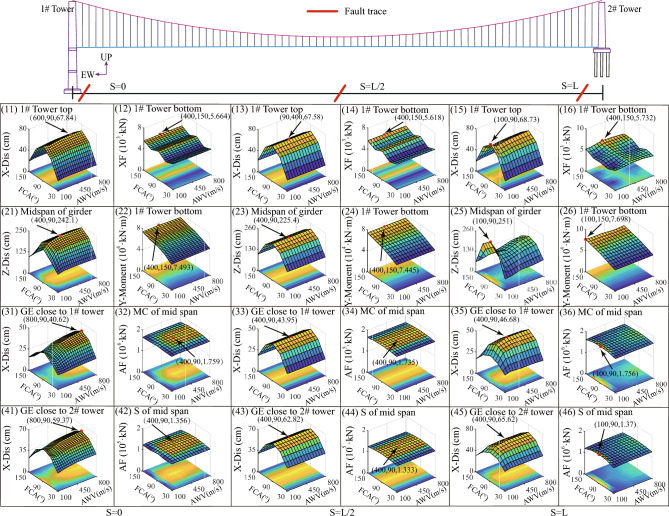
Response results in the Group B.

In order to offer a comprehensive understanding of the collective impact of the fault-crossing angle, location, and AWV on the responses of the long-span suspension bridge under seismic faulting, detailed 3D response diagrams are distinctly presented in Fig. 17. In Fig. 17, three fault rupture positions are examined. As indicated by the yellow area on the horizontal plane in Fig. 17, it suggests that two pivotal factors governing the responses of interest are low AWVs and an FCA (fault-crossing angle) of 90°. The peak displacements (X-Dis) on the top of the 1# tower in Fig. 17 (11), (13), and (15) measure 67.84 cm, 67.58 cm, and 68.73 cm, respectively. This indicates that the fault positions have minimal impact on the peak values and only alter the locations along the AWV axis where these peaks occur. The peak axial forces (XF) at the bottom of 1#tower in the Fig. 17 (12), (14), and (16) are 5.66 × 10^3^ kN, 5.62 × 10^3^ kN, and 5.73 × 10^3^ kN. The peak moments (Y-Moment) at the bottom of 1#tower in the Fig. 17 (22), (24), and (26) are 7.49 × 10^4^ kN m, 7.45 × 10^4^ kN m, and 7.70 × 10^4^ kN m. Likewise, there is nearly no discernible impact on other notable responses, such as the vertical displacement (Z-Dis) at the midspan of the girder, the longitudinal displacement (X-Dis) at the end of the girder close to the 1# tower, and the longitudinal displacement (X-Dis) at the end of the girder close to the 2# tower, by varying fault rupture locations. Consequently, it can be inferred that the fault rupture locations can be disregarded in the seismic design of the long-span suspension bridge.

### Effect of low, high, and broadband frequency (BF)

**Figure 18:**
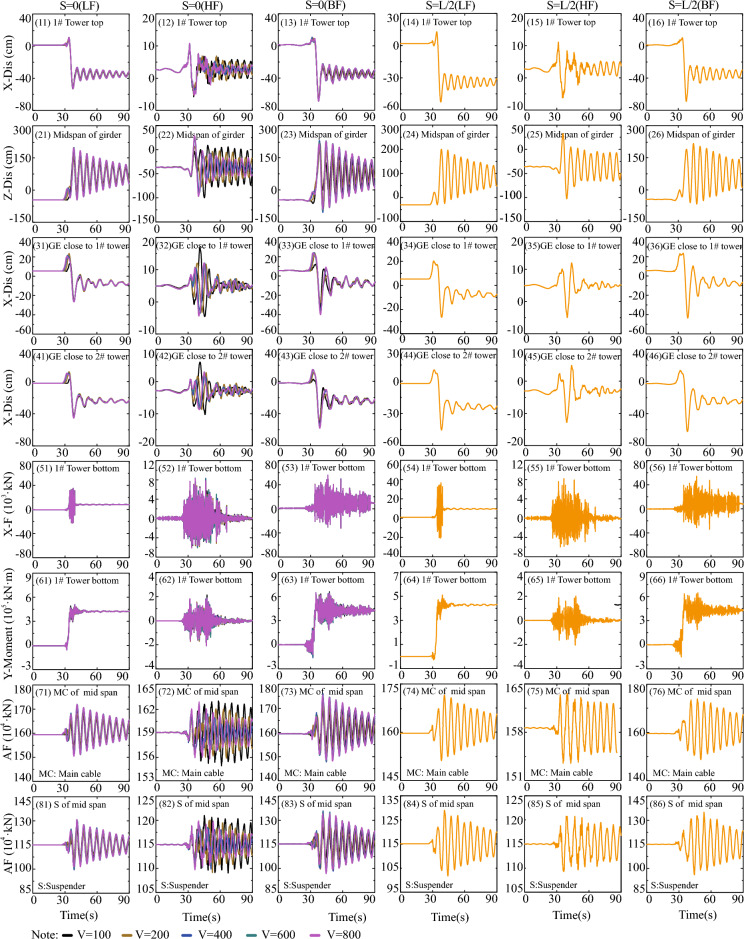
Comparison of LF, HF, and BF components in the Group C.

This section explores the influence of LF, HF, and BF components of ground motions across the fault on the responses of the long-span suspension bridge. The analysis of frequency components of the ground motions is confined within the fault rupture locations of S = 0 and S = L/2, as well as the fault-crossing angle of FCA = 90°, due to space limitations. Simultaneously, when the fault crosses through the center of the main span of the bridge (S = L/2), the arrival times of AWVs at both ends of the suspension bridge are the same, regardless of the AWVs. Consequently, there is no need to account for variations in AWVs, and only an orange line is displayed at the appropriate position in Fig. 18. As clearly observed in the top row of Fig. 18, the total displacement responses in Fig. 18 (13) and (16) at the top of the 1# tower are primarily influenced by the LF components displayed in Fig. 18 (11) and (14), rather than the HF components in Fig. 18 (12) and (15), respectively. In essence, it is the fault dislocation that generates LF pulse components that predominantly dictate the responses of the long-span suspension bridge spanning a fault. This pattern is similarly evident in Fig. 18, extending to internal force responses at the tower bottom and the displacement responses of the main girder. Consequently, it can be inferred that the LF components of ground motions across the fault play a central role in determining the internal force and displacement requirements of the long-span suspension bridge. Despite the flexibility of the long-span suspension bridge as a structural system, it remains a statically indeterminate structure. As illustrated in Fig. 18, LF ground motions result in higher internal force demands, while ground motions with HF components lead to comparatively smaller internal force requirements.

**Figure 19:**
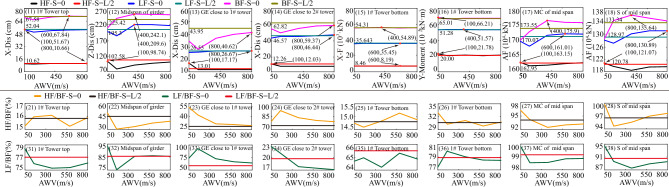
Proportion of LF and HF in BF components in the Group C.

To quantitatively measure the relative contributions of LF, HF, and BF components of the ground motions to the responses of the long-span suspension bridge, Fig. 19 displays the proportional relationships for various fault rupture positions and AWVs. Figure 19 (11) illustrates that the response lines for longitudinal displacement (X-Dis) of the tower top under LF ground motions are more akin to those under BF ground motions. The findings further confirm that LF components have a greater impact on the damage of the long-span suspension bridge across the fault, and the fault rupture locations and apparent wave velocities (AWVs) have minimal influence on the tower top displacement responses, as evidenced by the convergence of response lines of the same type. Similar trends are also observed in Fig. 19 (15) and (16). However, the responses of the main girder exhibit relatively significant dispersion, as shown in Fig. 19(13), (14), (17), and (18).

The ratio values of HF (HF) to broadband (BF) components are approximately 15% to 16.2% when considering the cases where S = 0 and S = L/2 in Fig. 19 (21). This implies that the responses of longitudinal displacement (X-Dis) of the tower top under HF ground motions constitute 15% to 16.2% of the total tower top responses. For the vertical displacement of the girder midspan in Fig. 19 (22), the ratio values of HF/BF are 45% when S = L/2 and range from 30 to 75% when S = 0. The distribution of the yellow line above the black line in Fig. 19 (23) and Fig. 19 (24) indicates that the HF components are significantly larger when S = 0 compared to when S = L/2. In Fig. 19 (25) and 19(26), the ratio values of HF/BF are less than 30% when S = L/2 and S = 0 respectively for the internal force responses of the tower bottom. The overall ratio values of LF/BF and HF/BF in Fig. 19 fall within the ranges of 60%-80% and 30%-40% respectively. Other specific details can be referred to in Fig. 19. Given the above-mentioned description and analysis, particular attention is devoted to the LF components of ground motions arising from the fling-step and directivity effects in the seismic design of long-span suspension bridges.

## Conclusions

The paper outlines a study that involves seismic and parametric response analyses of a fault-crossing suspension bridge. This analysis relies on a straightforward yet highly efficient method to generate the desired ground motions in close proximity to fault rupture planes. The accuracy and effectiveness of the presented method are confirmed through a comparison between the GGM and MGM. A comprehensive parametric analysis, encompassing factors such as fault-crossing location, crossing-fault angle, and the frequency components of across fault ground motions for the suspension bridge over a rupture fault, is performed using the sophisticated ANSYS software platform. The outcomes of this study have provided clear and specific specifications and guidelines for the seismic design of suspension bridges that traverse rupture faults. The following conclusions can be drawn from this investigation:

1. The method presented, which involves adding predicted faulting-induced surface rupture displacement due to the fling-step effect to processed ground motion time histories, is a straightforward and practical approach for seismic analysis of bridges spanning faults;
2. The accuracy and effectiveness of the method presented are validated through comparisons between generated ground motions (GGMs) and measured ground motions (MGMs), as well as by analyzing the responses of a long-span suspension bridge subjected to GGMs and MGMs, thereby fully meeting the seismic input requirements for analyzing bridges crossing dip-slip faults;
3. Overall, the responses of the long-span suspension bridge reach their maximum values when the fault-crossing angle (FCA) is set at 90°. In other words, an FCA of 90° represents the most unfavorable fault-crossing angle;
4. The locations of fault rupture and the AWVs can be neglected in the seismic design of the long-span suspension bridge with main span length of 538(m);
5. The LF components of across fault ground motions have primarily governed the seismic demands of the long-span suspension bridge across fault and the ratio values of LF/BF are in the ranges of 60%-80% approximately.

## Data Availability

The datasets used and/or analyzed during the current study available from the corresponding author on reasonable request.

## References

[1] Yang S, Mavroeidis GP (2018). Bridges crossing fault rupture zones: A review. Soil Dyn. Earthq. Eng..

[2] Ministry of Transport of the People' s Republic of China (MTPRC). Guideline for seismic design of highway bridges. Beijing, China (2020).

[3] European Committee for Standardization (CEN). Eurocode 8: Design of structures for earthquake resistance-part 1: general rules, seismic actions and rules for buildings (2004).

[4] Hongyu J, Jian Y, Shixiong Z, Canhui Z, Xiuli D (2021). A review on aseismic bridges crossing fault rupture regions. J. Southwest Jiaotong Univ..

[5] Lin Y, Li Y, Zong Z, Bi K, Xing K, Li Y (2023). Numerical study of seismic performance of steel-concrete composite rigid-frame bridge with precast segmental CFDST piers crossing fault-rupture zones. Structures..

[6] Shan J, Zhuang C, Cheng NL (2023). Parametric identification of Timoshenko-beam model for shear-wall structures using monitoring data. Mech. Syst. Signal Process..

[7] Shan J, Wang L, Cheng NL, Zhou Z (2023). Rapid seismic performance evaluation of existing frame structures using equivalent SDOF modeling and prior dynamic testing. J. Civil Struct. Health Monit..

[8] Jia H, Jia K, Sun C, Li Y, Zhang C, Zheng S (2021). Preliminary numerical study on seismic response of ordinary long-span suspension bridges crossing active faults. Adv. Bridge Eng..

[9] Zhang F, Li S, Wang J (2020). Effects of fault rupture on seismic responses of fault-crossing simply-supported highway bridges. Eng. Struct..

[10] Yang S, Mavroeidis GP, Tsopelas P (2021). Seismic response study of ordinary and isolated bridges crossing strike-slip fault rupture zones. Earthq. Eng. Struct. Dyn..

[11] Agalianos A, Sieber M, Anastasopoulos I (2020). Cost-effective analysis technique for the design of bridges against strike-slip faulting. Earthq. Eng. Struct. Dyn..

[12] Yuanzheng L, Zhouhong Z, Jin L, Yale Li, Yiyan C (2021). Across-fault ground motions and their effects on some bridges in the 1999 Chi-Chi earthquake. Adv. Bridge Eng..

[13] Fan Z, Shuai Li, Taiyi Z, Jingquan W (2021). Seismic cable restrainer design method to control the large-displacement response for multi-span simply supported bridges crossing fault rupture zones. Soil Dyn. Earthq. Eng..

[14] Lin Y, Zong Z, Bi K, Hao H, Lin J, Chen Y (2020). Experimental and numerical studies of the seismic behavior of a steelconcrete composite rigid-frame bridge subjected to the surface rupture at a thrust fault. Eng. Struct..

[15] Shuo Y (2022). Mavroeidis George P, Tsopelas Panos. Validation of the fault rupture-response spectrum analysis method for ordinary and seismically isolated bridges crossing strike-slip fault rupture zones. Eng. Struct..

[16] Goel RK, Chopra AK (2009). Nonlinear analysis of ordinary bridges crossing fault rupture zones. J. Bridge Eng..

[17] Goel RK, Chopra AK (2009). Linear analysis of ordinary bridges crossing fault-rupture zones. J. Bridge Eng..

[18] Goel RK, Chopra AK (2008). Role of shear keys in seismic behavior of bridges crossing fault-rupture zones. J. Bridge Eng..

[19] Goel R, Qu B, Tures J (2014). Validation of fault rupture-response spectrum analysis method for curved bridges crossing strike-slip fault rupture zones. J. Bridge Eng..

[20] Kamai R, Abrahamson N, Graves R (2014). Adding fling effects to processed ground-motion time histories. Bull. Seismol. Soc. Am..

[21] Abrahamson NA (2002). Velocity Pulses in Near-Fault Ground Motions.

[22] Tajammolian H, Khoshnoudian F, Loghman V (2017). Rotational components of near-fault earthquakes effects on triple concave friction pendulum base-isolated asymmetric structures. Eng. Struct..

[23] Graizer VM (2005). Effect of tilt on strong motion data processing. Soil Dyn. Earthq. Eng..

[24] Graizer V (2006). Tilts in strong ground motion. Bull. Seismol. Soc. Am..

[25] Lin Y, Zong Z, Tian S (2018). A new baseline correction method for near-fault strong-motion records based on the target final displacement. Soil Dyn. Earthq. Eng..

[26] Boore DM (2001). Effect of baseline corrections on displacements and response spectra for several recordings of the 1999 Chi-Chi, Taiwan, earthquake. Bull. Seismol. Soc. Am..

[27] Kamae K, Irikura K, Pitarka A (1998). A technique for simulating strong ground motion using hybrid Green's function. Bull. Seismol. Soc. Am..

[28] Ding Y, Mavroeidis GP, Theodoulidis NP (2019). Simulation of strong ground motion from the 1995 Mw 6.5 Kozani-Grevena, Greece, earthquake using a hybrid deterministic-stochastic approach. Soil Dyn. Earthq. Eng..

[29] Mavroeidis GP, Papageorgiou AS (2003). A mathematical representation of near-fault ground motions. Bull. Seismol. Soc. Am..

[30] Ucak A, Mavroeidis GP, Tsopelas P (2014). Behavior of a seismically isolated bridge crossing a fault rupture zone. Soil Dyn. Earthq. Eng..

[31] Nakano, T. & Ohta, Y. Non-linear dynamic response analysis of bridge crossing earthquake fault rupture plane. Beijing, China (2008).

[32] Li J (2020). Research on Single Tower Cable-Stayed Bridge's Seismic Response Crossing Faults.

[33] Yaguang Z (2016). Study on the Nonlinear Dynamic Response and Damage Feature of Cable-Stayed Bridge in Deep Water Excited by Cross—Fault Earthquakes.

[34] Yingxin H (2015). Study on Ground Motion Input and Seismic Response of Bridges Crossing Active Fault.

[35] Yitong Gu, Junjun G, Xinzhi D, Wancheng Y (2022). Seismic performance of a cable-stayed bridge crossing strike-slip faults. Structures.

[36] Goel RK (2008). Analysis of Ordinary Bridges Crossing Fault-Rupture Zones.

[37] Cole DA, Lade PV (1984). Influence zones in alluvium over dip-slip faults. J. Geotech. Eng..

[38] Saiidi MS, Vosooghi A, Choi H, Somerville P (2014). Shake table studies and analysis of a two-span RC bridge model subjected to a fault rupture. J. Bridge Eng..

[39] Yi J, Huaiyu Y, Jianzhong Li (2019). Experimental and numerical study on isolated simply-supported bridges subjected to a fault rupture. Soil Dyn. Earthq. Eng..

[40] Nailiang X, Huaiyu Y, Jianzhong Li (2019). Performance of an isolated simply supported bridge crossing fault rupture: shake table test. Earthq. Struct..

[41] Lin Y, Zong Z, Bi K, Hao H, Lin J, Chen Y (2020). Numerical study of the seismic performance and damage mitigation of steel–concrete composite rigid-frame bridge subjected to across-fault ground motions. Bull. Earthq. Eng..

[42] Yuanzheng L, Yiqian C, Zhouhong Z, Jin L, Guangwu T, Xiaohui He (2021). A new hybrid input strategy to reproduce across-fault ground motions on multi-shaking tables. J. Test. Evaluat..

[43] Park SW, Ghasemi H, Shen J, Somerville PG, Yen WP, Yashinsky M (2004). Simulation of the seismic performance of the Bolu Viaduct subjected to near-fault ground motions. Earthq. Eng. Struct. Dyn..

[44] Yang S, Mavroeidis GP, Ucak A (2017). Effect of ground motion filtering on the dynamic response of a seismically isolated bridge with and without fault crossing considerations. Soil Dyn. Earthq. Eng..

[45] Ge J, Saiidi MS (2018). Seismic response of the three-span bridge with innovative materials including fault-rupture effect. Shock Vib..

[46] Cong Z, Hui J, Xiaoyu B, Guangsong S (2023). Study on the multi-criteria seismic mitigation optimization of a single pylon cable-stayed bridge across strike-slip fault rupture zones. Engineering Structures..

[47] Jia H, Chen S, Guo D, Zheng S, Zhao C (2024). Track-bridge deformation relation and interaction of long-span railway suspension bridges subject to strike-slip faulting. Eng. Struct..

[48] Jia H, Chen S, Rui Wu, Guo D, Zhi Xu, Zheng S (2023). Response analyses of a hybrid cable-supported and sea-crossing bridge subjected to pulse-like ground motions and hydrodynamic force. Structures.

[49] Jia H, Liu Z, Li Xu, Bai H, Bi K, Zhang C, Zheng S (2023). Dynamic response analyses of long-span cable-stayed bridges subjected to pulse-type ground motions. Soil Dyn. Earthq. Eng..

[50] Byerly, P. & DeNoyer, J. Energy in earthquakes as computed from geodetic observations. In *Contributions in Geophysics in Honor of Beno Gutenberg* 17–35 (Pergamon Press, 1958).

[51] Hisada Y, Bielak J (2003). A theoretical method for computing near-fault ground motions in layered half-spaces considering static offset due to surface faulting, with a physical interpretation of fling step and rupture directivity. Bull. Seismol. Soc. Am..

[52] Xianlong He, Tianli S, Feng G (2016). A new method for picking up arrival times of seismic P and S waves automatically. Chin. J. Geophys. Chin. Ed..

[53] Makris N, Chang S-P (2000). Effect of viscous, viscoplastic and friction damping on the response of seismic isolated structures. Earthq. Eng. Struct. Dyn..

[54] Somerville PG (2003). Magnitude scaling of the near fault rupture directivity pulse. Phys. Earth Planet. Interiors.

[55] Infantino M, Smerzini C, Lin J (2021). Spatial correlation of broadband ground motions from physics-based numerical simulations. Earthq. Eng. Struct. Dyn..

